# Population heterogeneity in *Helicobacter pylori* PMSS1 shapes variable mouse infectivity: derivation of the homogeneous reference strain PMSS2

**DOI:** 10.1128/spectrum.01128-26

**Published:** 2026-09-17

**Authors:** Dowon Seo, Jaeyun Nam, Minseo Kim, Sungkyeong Lee, Jiwon Seo, Sacheera Angulmaduwa, Ashansa Ramanayake, Roshan Pradeep, Jing Lai, Eun-Jung Bak, Yong-Joon Cho, Eui-jeong Noh, Sungil Jang, Myeong-A Kim, Jeong-Heon Cha

**Affiliations:** 1 Department of Oral Biology, Oral Science Research Center, BK21 Four Project, Yonsei University College of Dentistry 92242, Seoul, Republic of Korea; 2 Department of Applied Life Science, The Graduate School, Yonsei University, Seoul, Republic of Korea; 3 Department of Periodontics, Changsha Stomatological Hospital 478929 , Changsha, Hunan, China; 4 Department of Molecular Bioscience, Kangwon National University 34962, Chuncheon, Republic of Korea; 5 Multidimensional Genomics Research Center, Kangwon National University 34962 , Chuncheon, Republic of Korea; Griffith University - Gold Coast Campus, Gold Coast, Queensland, Australia

**Keywords:** *Helicobacter pylori*, PMSS1, PMSS2, population heterogeneity, whole-genome sequencing, mouse infectivity, host-pathogen interaction

## Abstract

Experimental infection models are widely used to investigate host–microbe interactions, often under the assumption that bacterial populations are genetically uniform. Here, we examined population heterogeneity in the widely used *Helicobacter pylori* strain PMSS1 and its relationship to variation in mouse infectivity. Single-colony isolates derived from PMSS1 displayed substantial differences in colonization efficiency, indicating that pre-existing variation within the population contributes to infection outcomes. To distinguish the effects of initial population heterogeneity from changes arising during infection, we analyzed PMSS2, a genetically homogeneous reference strain derived from PMSS1 that exhibited consistent infection phenotypes across independently isolated clones. Comparative genomic analysis of isolates recovered from infected mice revealed differences in the extent and patterns of genomic variation between PMSS1- and PMSS2-derived populations. These results demonstrate that variability in infection outcomes can arise from pre-existing heterogeneity within bacterial populations and highlight the importance of considering population composition when interpreting experimental infection studies.

**IMPORTANCE:**

Animal infection models are widely used to study how bacterial pathogens cause disease and change during infection. These studies often assume that the bacteria used for infection are genetically uniform. Our study shows that this assumption may not always hold. We found that a commonly used *Helicobacter pylori* strain contains hidden genetic diversity that leads to large differences in how well bacteria infect mice. By comparing this strain with a genetically uniform derivative, we show how differences present before infection can shape infection outcomes and influence the genetic changes observed during infection. Our findings highlight the importance of considering starting population diversity when interpreting experimental infection studies and are broadly relevant to research on microbial pathogenesis.

## INTRODUCTION

*Helicobacter pylori* is a highly diverse bacterial pathogen that establishes chronic infection in the human stomach and can persist for decades within its host (1–4). This long-term association is accompanied by substantial genetic variability, which has been extensively investigated in the context of host adaptation, immune pressure, and disease progression (5–9). Experimental infection models, particularly mouse models, have played a central role in advancing our understanding of these host–pathogen interactions (10–14).

Among *H. pylori* strains used for experimental infection, PMSS1 has been widely adopted as a mouse-colonizing reference strain because of its ability to establish persistent infection in C57BL/6J mice and support functional type IV secretion system activity (15, 16). As a result, PMSS1 has served as the foundation for numerous studies examining bacterial virulence, host immune responses, and infection-associated genetic changes *in vivo* (11, 12, 17, 18).

Experimental infection studies typically rely on laboratory-maintained bacterial stocks and often proceed under the practical assumption that these stocks represent a consistent starting population for *in vivo* analysis, frequently following single-colony isolation (12, 19). In practice, bacterial populations propagated under routine laboratory conditions can accumulate genetic variation over time, raising the possibility that individual cells within a stock may differ in their genomic composition prior to infection (5, 6, 18, 20–22). Such pre-existing variation could influence phenotypic outcomes observed in infection experiments and complicate the interpretation of genomic changes detected after host colonization (9, 23, 24).

Several lines of prior work suggest that PMSS1 may not represent a genetically uniform entity (15, 24–26). Genome-based analyses have shown that individual PMSS1 chromosomes frequently harbor multiple tandem copies of the *cagA* virulence gene, indicating structural complexity at the single-genome level (15, 25). At the same time, population-based assays have demonstrated that PMSS1 stocks can comprise bacterial subpopulations with differing *cagA* copy numbers, revealing heterogeneity at the population level. These observations raise an important conceptual issue: genomic features inferred from bulk or single-genome analyses do not necessarily capture the full extent of diversity present within a bacterial population, nor do they clarify how such diversity may be reflected in phenotypic outcomes observed during infection.

In this study, we examine the genomic composition of a commonly used PMSS1 laboratory stock and assess how intrinsic population structure influences infection outcomes in mice. By combining whole-genome sequencing, single-colony infection assays, and longitudinal analysis of infection-associated genomic changes, we demonstrate that PMSS1 comprises a genetically heterogeneous population associated with variable mouse infectivity. We further describe PMSS2, a genetically homogeneous derivative of PMSS1, which exhibits highly reproducible infection phenotypes. Using this strain as a homogeneous reference, we distinguish genomic variation arising during host infection from the pre-existing population heterogeneity present in PMSS1. Together, our findings provide insight into how pre-existing population heterogeneity influences infection outcomes and the interpretation of genomic variation in experimental *H. pylori* models.

## RESULTS

### PMSS1 laboratory stock exhibits intrinsic genomic heterogeneity associated with variable mouse infectivity

**Fig 1:**
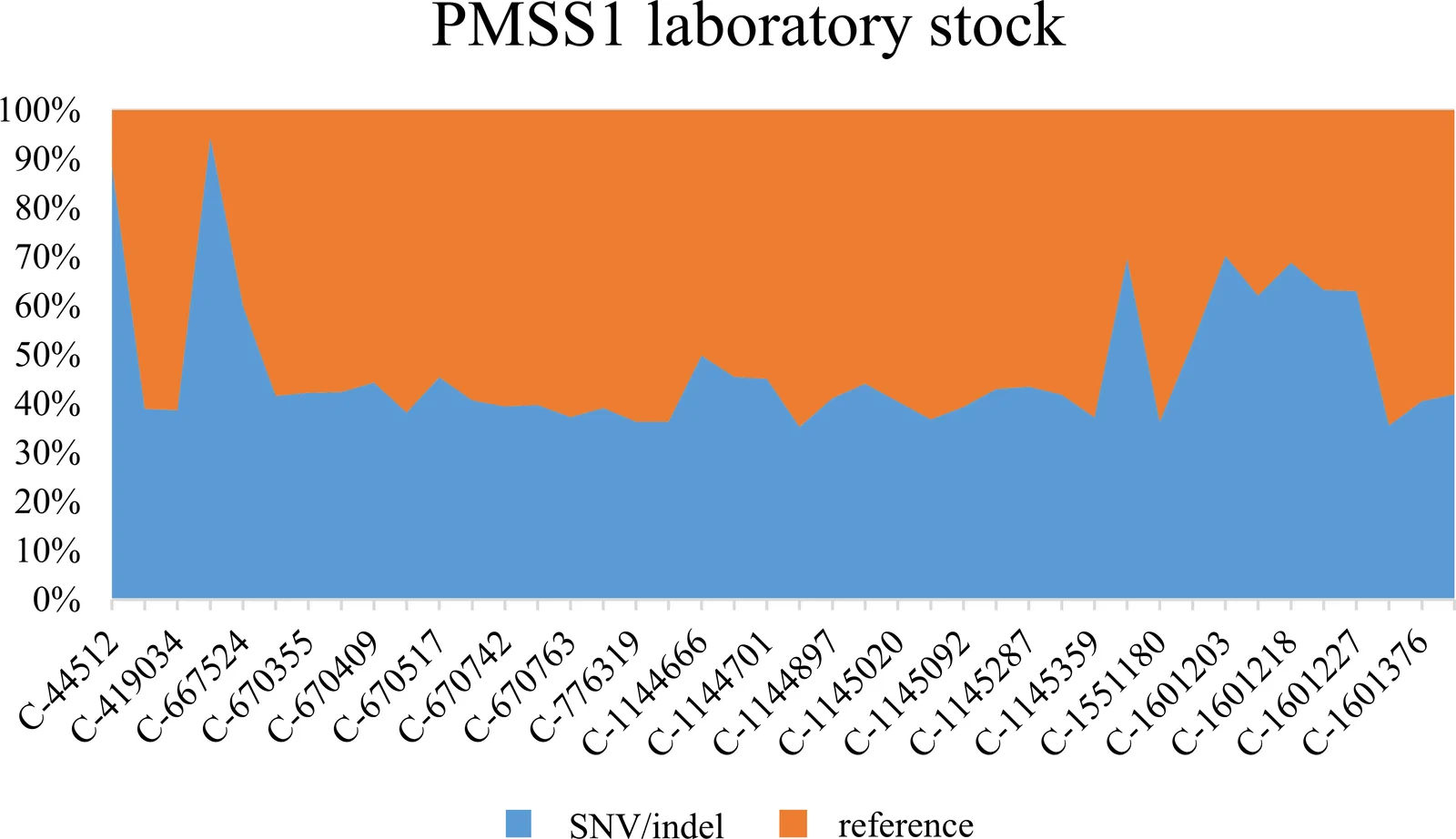
Genomic heterogeneity within the PMSS1 laboratory stock. Whole-genome sequence comparison between the PMSS1 laboratory stock and the PMSS1 reference genome revealed a total of 41 SNVs and small indels distributed across the genome. At each of these positions, the allele-frequency composition is shown as the proportion of variant (SNV/indel) vs reference alleles. The average proportion of variant alleles across these positions was approximately 47.4%, indicating that the PMSS1 laboratory stock represents a genetically heterogeneous population rather than a clonal strain. The mean sequencing depth of the laboratory stock sample was approximately 1,010×, yielding an effective allele frequency detection threshold of approximately 5% (see Table S5 for the full depth summary of all sequenced samples).

PMSS1 is widely used as a mouse-colonizing *H. pylori* strain; however, the genetic composition of commonly used laboratory stocks has not been systematically evaluated. Whole-genome sequencing of the PMSS1 stock used in this study revealed multiple single nucleotide variants (SNVs) and small insertions/deletions (indels) present at intermediate allele frequencies, indicating that the stock represents a mixed population rather than a clonal entity (Fig. 1).

**Fig 2:**
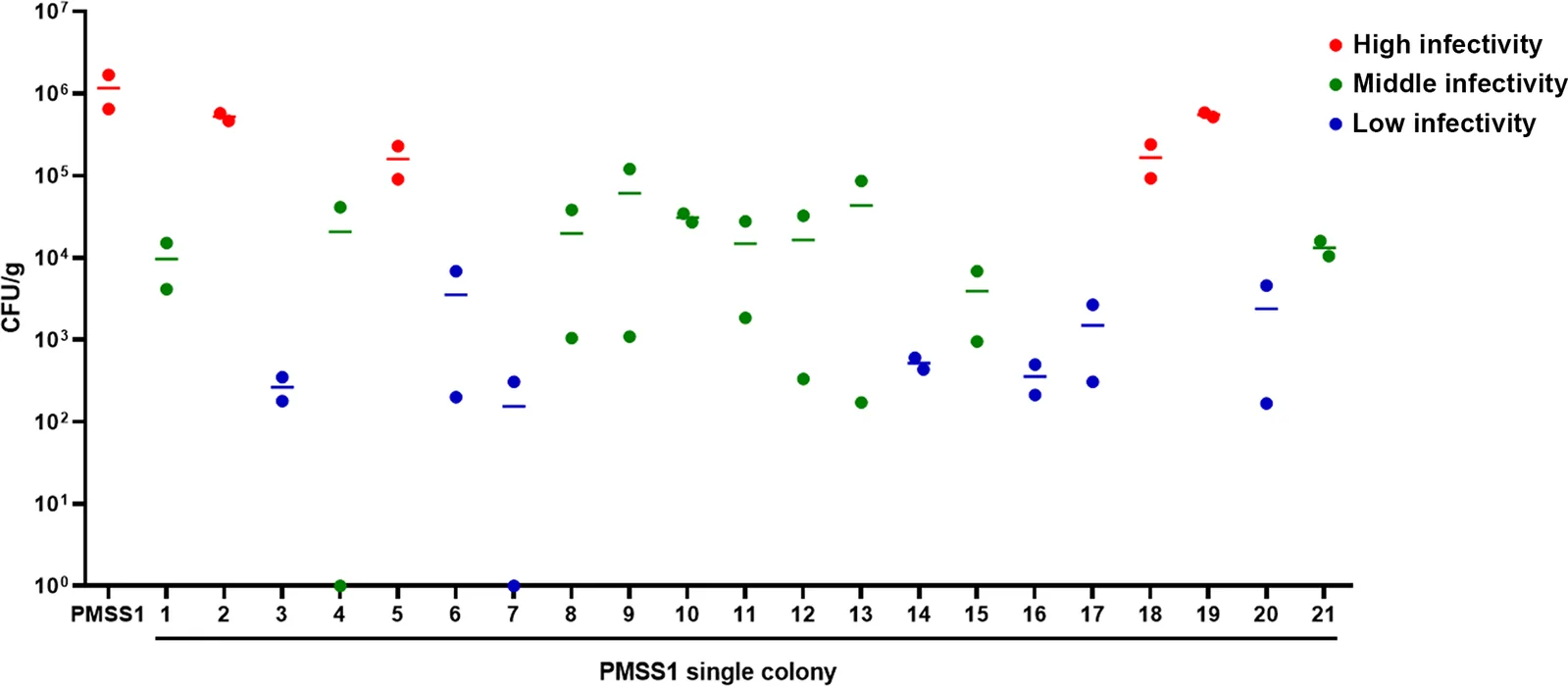
Variable mouse infectivity among PMSS1-derived single colonies. Twenty-one single colonies were isolated from the *H. pylori* PMSS1 strain and used to infect C57BL/6J mice for 1 week. Two mice were inoculated per single colony, and each data point represents the colony-forming units (CFU) per gram of stomach tissue recovered from an individual mouse; short horizontal bars indicate group medians. Isolates were classified into high-, middle-, and low-infectivity groups based on their median CFU/g values relative to the parental PMSS1 stock (see Statistical analysis in Materials and Methods for cutoff values). Substantial variability in colonization levels was observed among PMSS1-derived single colonies, as assessed by CFU recovered from mouse stomachs. Comparisons among the three infectivity groups were performed using the Kruskal–Wallis test followed by Dunn’s multiple comparisons test.

To determine whether this intrinsic genomic heterogeneity is reflected at the phenotypic level, single colonies were isolated from the PMSS1 stock and individually tested for their ability to colonize the murine stomach (Fig. S1). Following short-term infection, PMSS1-derived single colonies displayed marked variability in colonization levels, ranging from near-undetectable to robust colonization (Fig. 2). Statistical analysis confirmed significant differences among the three infectivity groups (Kruskal–Wallis *H* = 25.53, adjusted *P* = 2.9 × 10^−6^), with all pairwise comparisons reaching statistical significance (high vs middle: adjusted *P* = 7.7 × 10^−6^; high vs low: adjusted *P* = 4.5 × 10^−4^; and middle vs low: adjusted *P* = 3.6 × 10^−3^). These results demonstrate that differences in mouse infectivity pre-exist within the PMSS1 laboratory stock.

### Genome sequencing of PMSS1-derived single colonies reveals diverse genetic architectures

**Fig 3:**
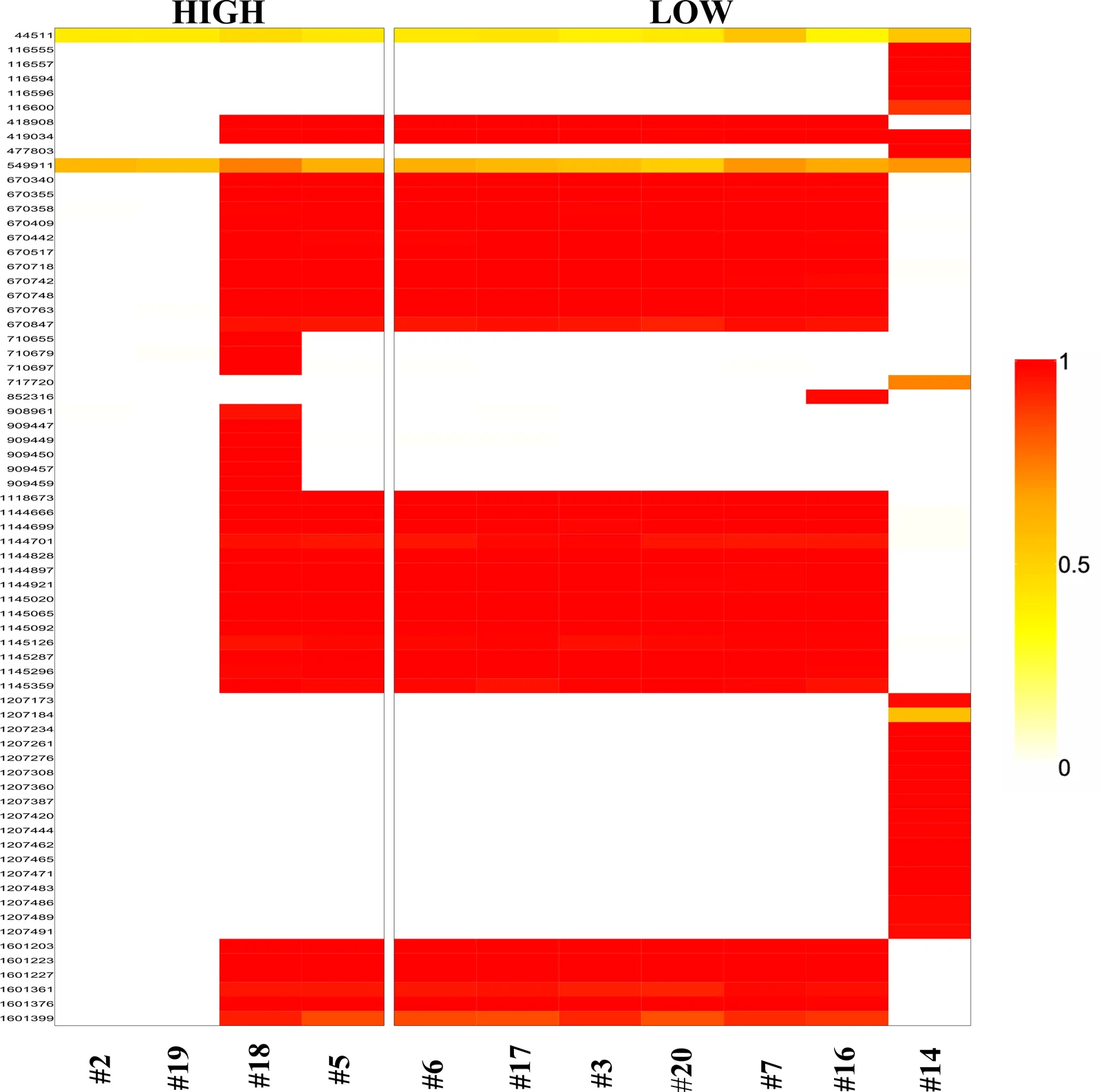
Genome sequence variation in a subset of PMSS1-derived single colonies. Whole-genome sequencing was performed on a phenotype-driven subset of 11 PMSS1-derived single colonies representing high- and low-infectivity groups. Genome sequences were compared to the PMSS1 reference genome. The heatmap displays the allele frequency of each variant in each isolate, with color intensity indicating allele frequency: darker red indicates higher allele frequency (approaching 100% in fixed variants), lighter shades indicate lower allele frequencies, and white indicates absence of the variant.

To assess the genomic diversity associated with variability in infectivity, whole-genome sequencing was performed on selected PMSS1-derived single colonies representing high- and low-infectivity phenotypes. Comparative analysis against the PMSS1 reference genome revealed substantial sequence divergence among most sequenced colonies, including numerous SNVs and indels distributed across the genome (Fig. 3). In contrast, two isolates (#2 and #19) with high-infectivity phenotypes exhibited minimal sequence divergence and retained near-reference-like genomic profiles despite originating from the heterogeneous PMSS1 population.

Consistent with these findings, analysis of non-synonymous SNVs identified across the sequenced isolates revealed a broad spectrum of genetic changes affecting diverse functional categories (Table 1), underscoring the genomic diversity that can arise among single colonies derived from a single laboratory strain.

**TABLE 1:** Distribution of non-synonymous variants across isolates grouped by infectivity^*a*^

POS	Gene (locus tag)	Variant	High infectivity					Low infectivity					
			#2	#19	#18	#5	#6	#17	#3	#20	#7	#16	#14
44511	HPYLPMSS1_00046	Frameshift	●	●	●	●	●	●	●	●	●	●	●
418908	*fur* (HPYLPMSS1_00405)	R87Q			●	●	●	●	●	●	●	●	
419034	*fur* (HPYLPMSS1_00405)	H45P			●	●	●	●	●	●	●	●	●
477803	*katA* (HPYLPMSS1_00459)	C361F											●
908961	*futB* (HPYLPMSS1_00858)	N221K			●								
909447	*futB* (HPYLPMSS1_00858)	L59F			●								
909457	*futB* (HPYLPMSS1_00858)	T56K			●								
1144701	HPYLPMSS1_01072	T161A			●	●	●	●	●	●	●	●	
1144921	HPYLPMSS1_01072	D87E			●	●	●	●	●	●	●	●	
1145126	HPYLPMSS1_01072	V19A			●	●	●	●	●	●	●	●	
1207491	*sotB* (HPYLPMSS1_01131)	I302V											●
1601203	HPYLPMSS1_01511	F3S			●	●	●	●	●	●	●	●	
1601227	*engB* (HPYLPMSS1_01512)	L208S			●	●	●	●	●	●	●	●	
1601361	*engB* (HPYLPMSS1_01512)	D163E			●	●	●	●	●	●	●	●	
1601399	*engB* (HPYLPMSS1_01512)	S151T			●	●	●	●	●	●	●	●	

^*a*^ Non-synonymous variants relative to the PMSS1 reference genome are listed, and the presence of each variant in individual isolates is indicated by a filled circle (●). Variants recurring across multiple isolates within the same infectivity group highlight intrinsic genetic heterogeneity within the PMSS1 population.

### Allele frequency patterns distinguish PMSS1-derived single colonies from the parental stock

**Fig 4:**
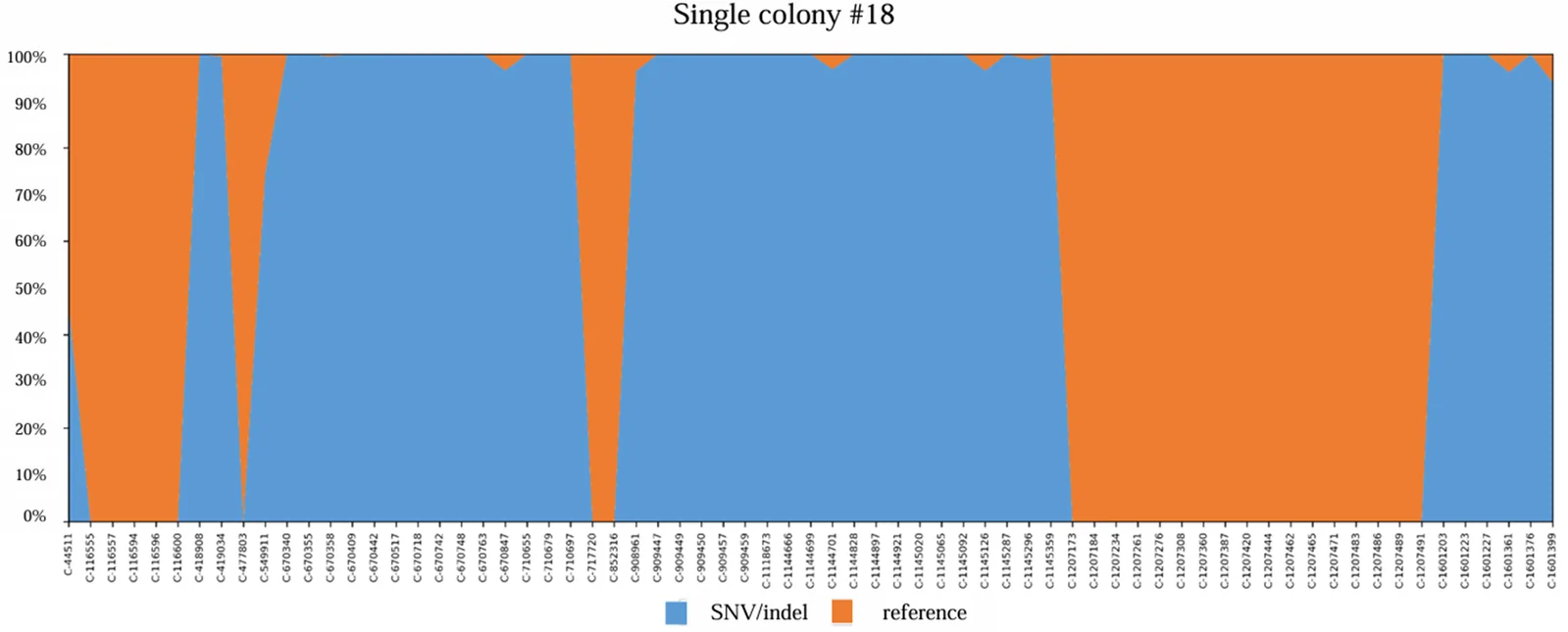
Allele frequency patterns reveal mixed population structure in PMSS1. The percentage of variant alleles at each SNV/indel position was analyzed for representative PMSS1-derived single-colony isolate #18, which showed a high average variant allele frequency of 97.6% across 44 variant positions. Similar allele frequency patterns were observed in the majority of PMSS1-derived single colonies subjected to whole-genome sequencing.

To further characterize the genomic composition of individual PMSS1-derived colonies, variant allele frequencies were examined at SNV and indel positions in representative isolates. Isolate #18 exhibited a high average variant allele frequency (AF) (97.6%) across 44 variant positions, indicating that the majority of detected variants were fixed within this clone (Fig. 4). Similar allele frequency patterns were observed across most PMSS1-derived single colonies subjected to whole-genome sequencing. In contrast, the parental PMSS1 stock displayed intermediate allele frequencies across many positions, consistent with a mixed population structure.

To further assess the genomic heterogeneity of PMSS1, *cagA* copy number was quantified in the parental stocks, single-colony isolates, and 4-week mouse output colonies using two orthogonal approaches: whole-genome sequencing depth-based estimation and quantitative real-time PCR—which yielded strongly concordant estimates (Pearson *r* = 0.955, *P* = 4.7 × 10^−6^, *n* = 11; Fig. S2). PMSS1-derived single colonies exhibited a broad distribution of *cagA* copy numbers (quantitative real-time PCR [qPCR]-based: 1.9–4.1 copies; WGS depth-based: 1.9–3.9 copies), consistent with structural heterogeneity of the parental stock.

### Mouse infectivity phenotypes of PMSS1-derived isolates are not fixed across infection cycles

Given the pronounced variability in infectivity observed among PMSS1-derived single colonies, we next examined whether these phenotypes were maintained across successive infection cycles. Output isolates recovered from primary infection experiments were re-tested in independent mouse infection assays. Isolates that initially exhibited low infectivity (#3 and #7) showed increased colonization levels upon re-infection (Fig. S3), indicating that reduced infectivity was not stably maintained across infection cycles. In contrast, isolate #2, which initially displayed a high-infectivity phenotype, retained robust colonization capacity upon re-infection.

### Identification of PMSS2 as a genetically homogeneous derivative with stable infectivity

**Fig 5:**
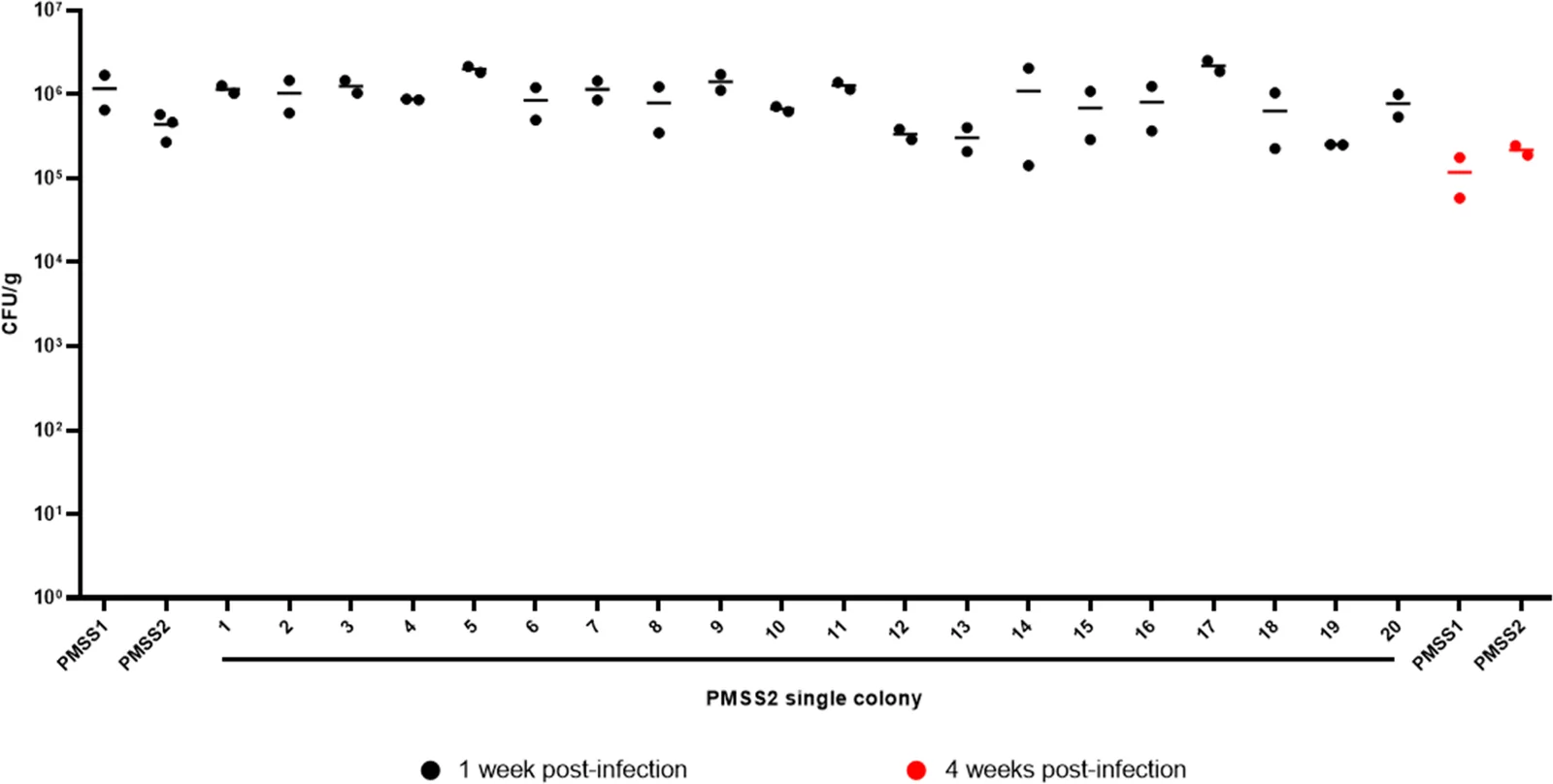
Stable infectivity of PMSS2-derived single colonies during mouse infection. C57BL/6J mice were orally infected with the PMSS2 strain or with 20 single colonies isolated from PMSS2. Bacterial colonization levels were determined by enumerating CFU/g of stomach tissue at 1 week (black) or 4 weeks (red) post-infection. Two mice were inoculated per bacterial group (three mice for the parental PMSS2 group at 1 week post-infection), and each data point represents CFU/g of stomach tissue recovered from an individual mouse. Data for the PMSS1 and PMSS2 strains are shown for comparison. Pairwise comparisons between the parental PMSS2 strain and the PMSS2-derived single-colony group at 1 week post-infection were performed using the Mann–Whitney *U* test. Comparisons between 1- and 4-week time points for PMSS1 and PMSS2 were performed using two-way ANOVA on log_10_-transformed CFU/g values with Sidak’s multiple comparisons test.

During characterization of PMSS1-derived single colonies, we identified a derivative isolate (#2) whose genome sequence differed from the PMSS1 reference at only two single nucleotide positions and exhibited high infectivity upon re-infection. This isolate, designated PMSS2, was used to generate independently isolated single colonies for infection experiments. In contrast to PMSS1-derived isolates, PMSS2-derived single colonies exhibited highly consistent colonization levels in mice following short-term infection (Fig. 5). At 1 week post-infection, PMSS2-derived single-colony isolates showed comparable colonization levels to the parental PMSS2 strain (median colony-forming unit [CFU]/g: 1,013,889 vs 466,667; Mann–Whitney *U*, *P* = 0.146). PMSS2 infection also resulted in reproducible colonization patterns over a prolonged 4-week infection period, indicating a stable infectivity phenotype across independently isolated clones. Comparing 1- and 4-week time points, PMSS1 colonization declined significantly (*P* = 0.0419), whereas PMSS2 colonization remained stable (*P* = 0.7969), consistent with the greater colonization stability observed for PMSS2.

### Distinct genetic distance patterns emerge during mouse infection with PMSS1 and PMSS2

**Fig 6:**
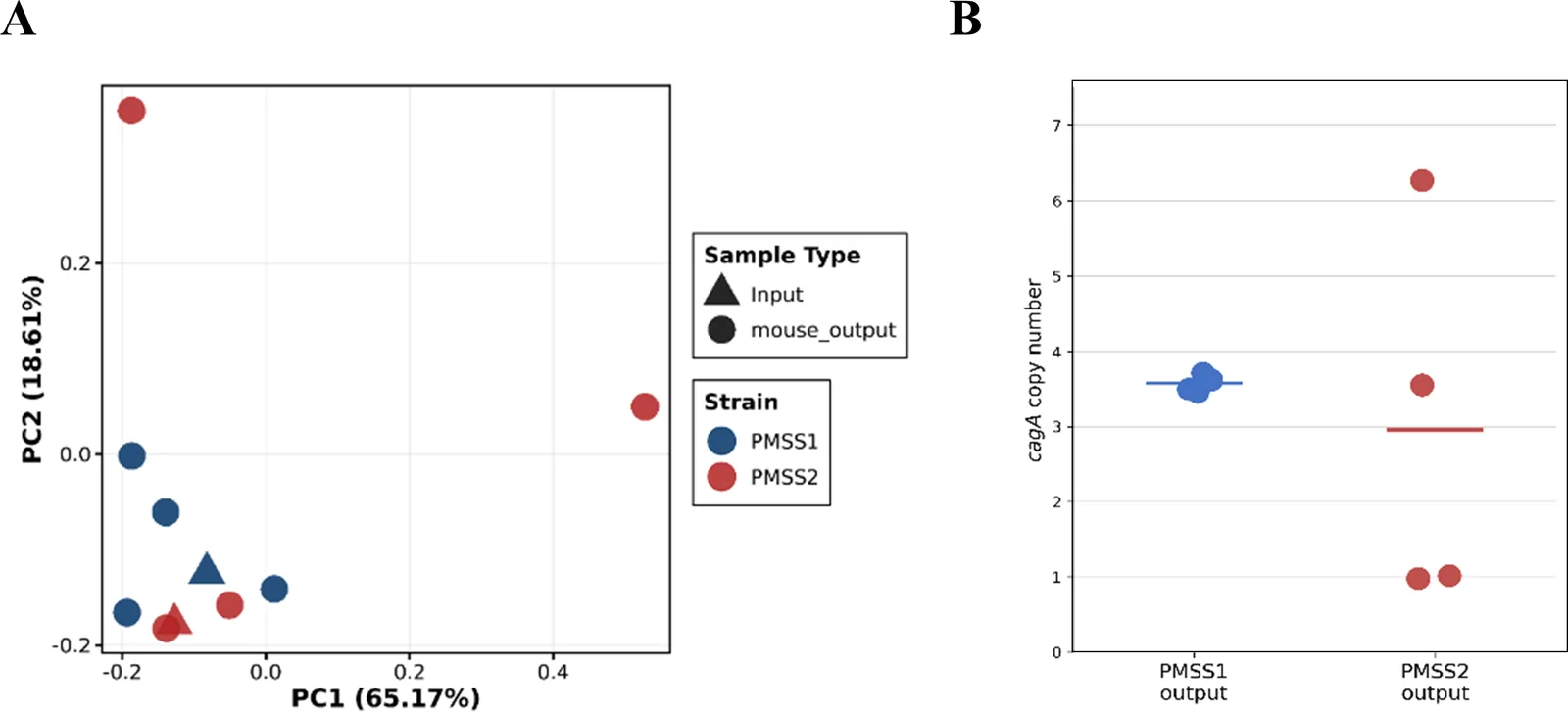
Distinct genetic distance patterns of PMSS1 and PMSS2 following mouse infection. (**A**) Principal component analysis (PCA) was performed to visualize the genetic distances between the initial input populations and the single colonies recovered after 4 weeks of mouse infection. PC1 explains 65.17% and PC2 explains 18.61% of the total variance in genome-wide variant profiles. Triangles denote the initial input populations of PMSS1 and PMSS2; circles denote single colonies recovered from individual mice 4 weeks post-infection. For PMSS1 (blue), the mouse-output colonies remained closely clustered with the starting input population (triangle), indicating relatively conserved genomic stability and limited divergence during colonization. In striking contrast, PMSS2 (red) output colonies exhibited significant genetic dispersion from their respective input, shifting along distinct axes (PC1 and PC2). These results demonstrate that PMSS2 undergoes more extensive and diverse evolutionary diversification within the host environment compared to the relatively stable PMSS1 strain. (**B**) *cagA* copy number of individual single colonies recovered from mice 4 weeks post-infection with PMSS1 or PMSS2, quantified by whole-genome sequencing depth-based estimation. PMSS1-derived output colonies converged to a narrow range (3.46–3.71 copies; mean 3.57), whereas PMSS2-derived output colonies showed wide dispersion (0.98–6.27 copies; mean 2.96), consistent with divergence during *in vivo* colonization.

To examine genomic changes arising during prolonged infection, whole-genome sequencing was performed on single colonies isolated from individual mice after 4 weeks of infection with PMSS1 or PMSS2. PMSS1-derived single colonies recovered from the same mouse exhibited relatively similar genetic distances to one another, indicating limited divergence among colonies following infection (Fig. 6A). In contrast, PMSS2-derived single colonies displayed greater variability in genetic distances, with some colonies diverging along distinct genetic trajectories (Fig. 6A). This pattern was recapitulated at the level of structural variation: *cagA* copy number in PMSS1-derived output colonies converged to a narrow range (3.46–3.71 copies; mean 3.57), whereas PMSS2-derived output colonies exhibited wide dispersion (0.98–6.27 copies; mean 2.96), paralleling the divergence pattern observed at the SNV level (Fig. 6B).

These opposite patterns reflect fundamentally different evolutionary trajectories of heterogeneous vs clonal starting populations. In PMSS1, the initial input population is already heterogeneous, and prolonged colonization drives the population toward a common subset of dominant variants, producing convergence of output clones in genetic distance space. In PMSS2, the initial input population is nearly clonal, so mutations that arise during infection accumulate along independent trajectories in different mice and different clones, producing divergence.

### Aggregated and pooled genome sequencing reveals differential mutational outputs following infection

**Fig 7:**
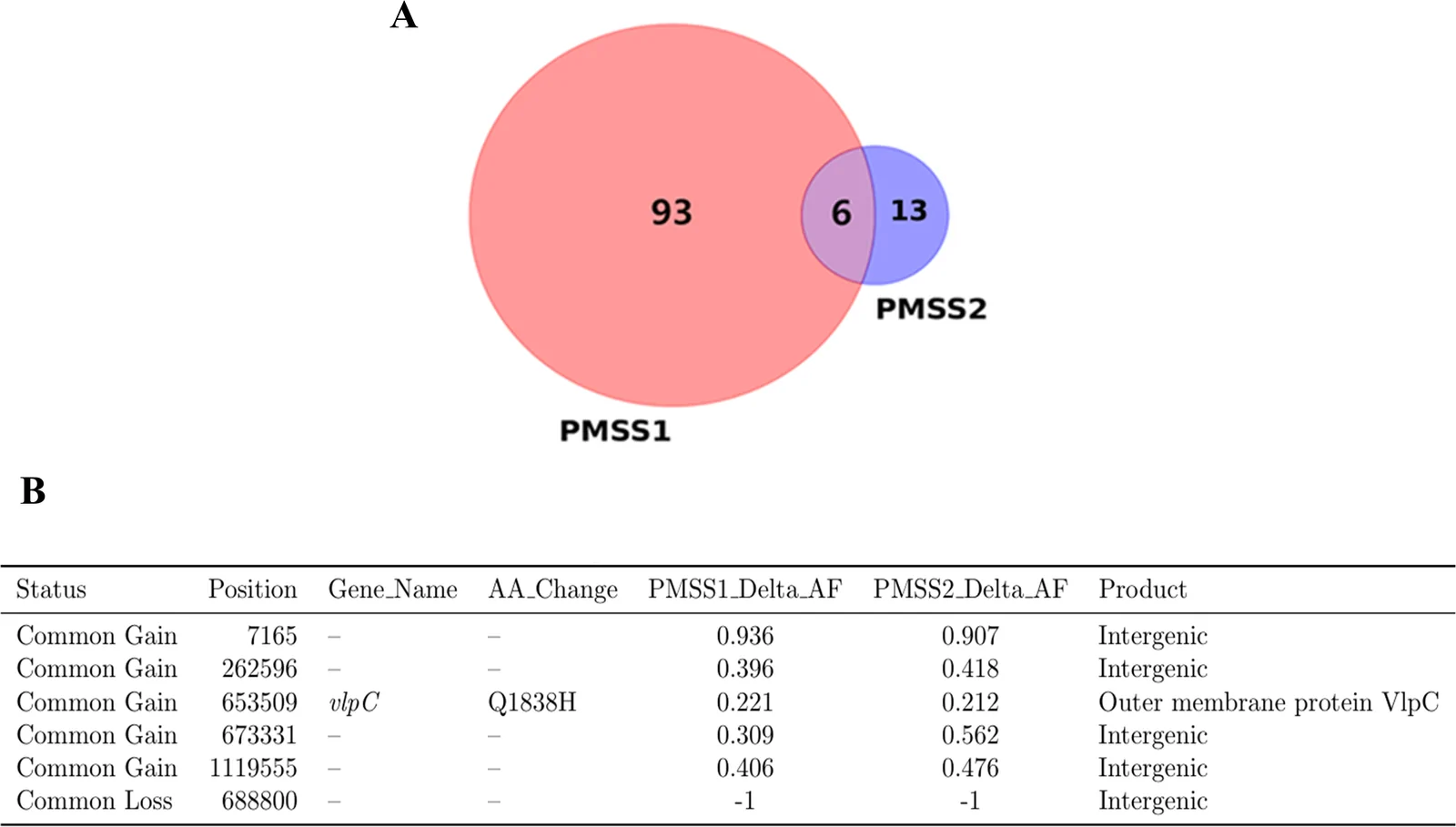
Aggregated mutational outputs of PMSS1 and PMSS2 following 4 weeks of mouse infection. Whole-genome sequencing was performed on pooled bacterial populations generated by aggregating all output single colonies recovered after 4 weeks of mouse infection with PMSS1 or PMSS2. (**A**) The total number of mutations identified in each population is shown. PMSS1 yielded a substantially higher number of mutations (99 total mutations) compared to PMSS2 (19 total mutations). Venn diagram showing mutations shared between PMSS1 and PMSS2. Six mutations were identified in common, representing overlapping genetic changes detected following host colonization. (**B**) The six mutations identified in common between PMSS1- and PMSS2-derived pooled output populations (corresponding to the overlap shown in panel **A**) are listed, including genomic position, gene name (where applicable), amino acid change, the change in allele frequency (ΔAF) in each population, and the predicted product.

To assess population-level genomic changes following prolonged infection, output single colonies recovered after 4 weeks were aggregated and subjected to whole-genome sequencing. Analysis of aggregated mutational outputs revealed that PMSS1 accumulated a substantially higher number of mutations (99 total mutations) compared to PMSS2 (19 total mutations) (Fig. 7; Fig. S4). Six mutations were shared between PMSS1- and PMSS2-derived output populations, representing overlapping genetic changes detected following host colonization.

Given that the PMSS1 population was initially heterogeneous, pooled allele frequency analysis revealed how prolonged gastric colonization selectively reshaped the population composition. Following 4 weeks of mouse infection, only a subset of variants increased in frequency, indicating preferential survival and expansion of bacterial subpopulations better adapted to the gastric environment, while other lineages declined or were eliminated.

**Fig 8:**
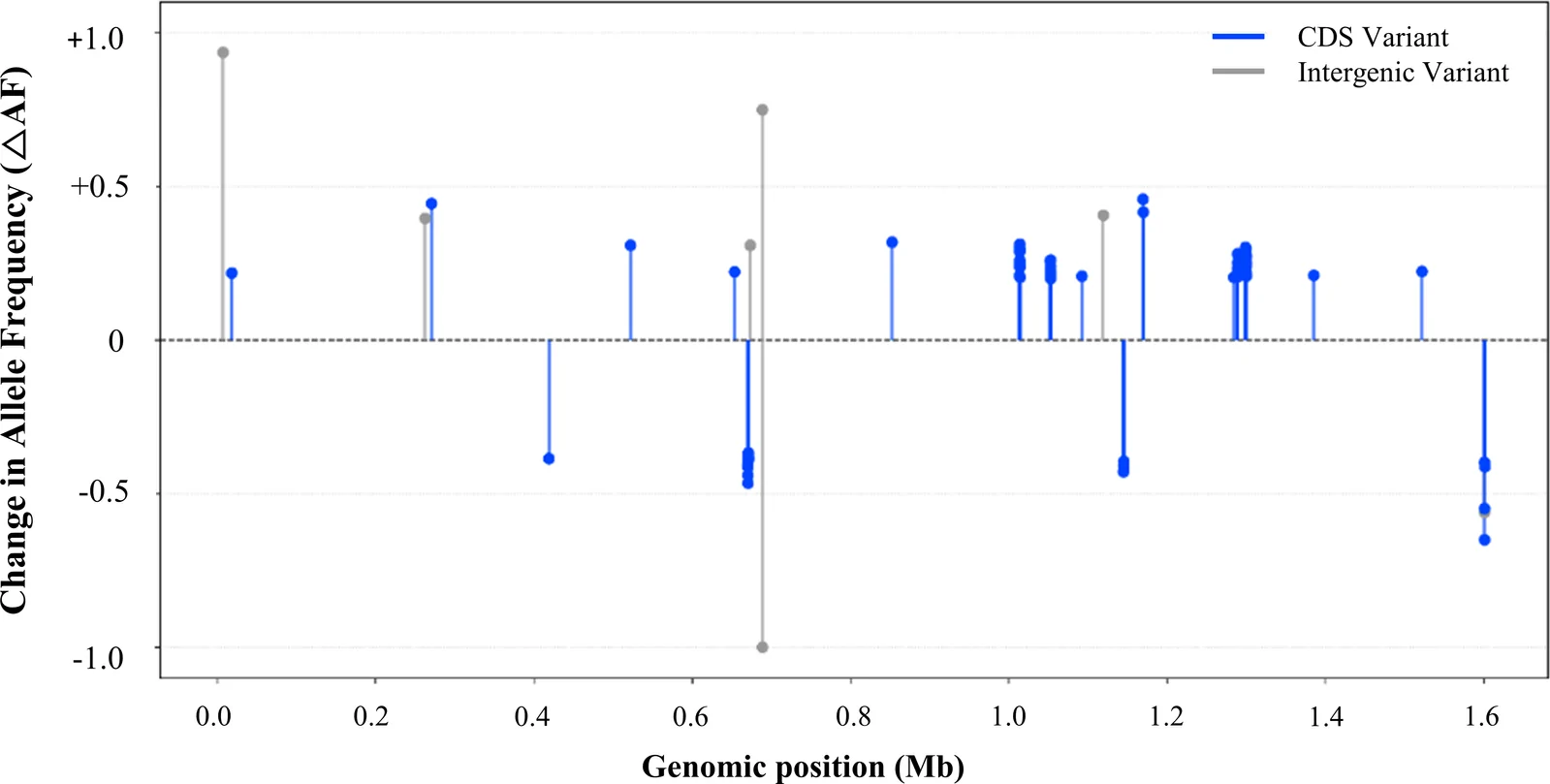
Genome sequencing of pooled output colonies recovered after mouse infection. Pooled population analysis revealed the allele frequency dynamics between the initial PMSS1 heterogeneous input pool and the pooled output population recovered after 4 weeks of mouse infection. The lollipop plot visualizes the change in allele frequency (ΔAF) for detected variants across the *H. pylori* genome. Positive ΔAF values (upward lollipops) indicate clonal expansion, where specific variants gained dominance in the population, while negative ΔAF values (downward lollipops) represent lineage loss, indicating variants that decreased in frequency or were eliminated. Variants are color-coded based on their genomic location: coding sequence (CDS) variants are in blue, and intergenic variants are in gray. Complete gene-level annotations for all variants shown are provided in Table S1, Sheet 3 (lollipop).

These allele frequency shifts were non-randomly distributed across the genome and affected both coding sequence and intergenic variants, suggesting that host-driven selective pressures act on specific genetic loci to refine the initially heterogeneous population during *in vivo* colonization (Fig. 8).

## DISCUSSION

In this study, we investigated the genomic and phenotypic consequences of intrinsic strain heterogeneity during *H. pylori* infection using the widely employed mouse-colonizing strain PMSS1 and a newly identified derivative, PMSS2. Our findings demonstrate that commonly used PMSS1 laboratory stocks comprise a genetically heterogeneous population associated with marked variability in mouse infectivity, and that this heterogeneity fundamentally influences infection outcomes and the interpretation of host-associated genomic changes.

A critical conceptual distinction emerging from this work is the difference between multi-copy genomic architecture and population-level heterogeneity. Previous studies, including our own, have shown that PMSS1 genomes frequently harbor multiple tandem copies of *cagA*, a feature that can be resolved by qPCR and long-read genome sequencing and reflects structural properties of individual chromosomes (9, 15, 25–27). However, the presence of a multi-*cagA* genotype alone does not constitute evidence of population heterogeneity. Rather, heterogeneity at the population level—defined by the coexistence of bacterial cells with differing *cagA* copy numbers—can be directly demonstrated only by assays capable of resolving mixed genomic states, such as Southern blot analysis (15, 25, 28, 29). Notably, such *cagA* copy number heterogeneity has been reproducibly observed in PMSS1 stocks derived from independent laboratory sources and characterized using distinct experimental approaches, indicating that population-level heterogeneity represents an intrinsic and stable property of PMSS1 rather than a stock-specific or methodological artifact (15, 25, 29).

Our results further show that genomic heterogeneity within PMSS1 laboratory stocks is accompanied by substantial phenotypic variability in mouse infectivity at the level of single colonies. Importantly, this variability pre-exists prior to host infection, suggesting that pre-existing population variation represents one source of variability contributing to inconsistent infection outcomes, alongside host-related factors, stochastic bottleneck effects during colonization, and potential *in vivo* adaptive processes. From this perspective, the heterogeneous infection outcomes observed among PMSS1-derived isolates need not be interpreted as evidence of active host-driven diversification but rather as differential expression of genomic variation accumulated during routine *in vitro* cultivation—an unavoidable feature of long-term laboratory strain maintenance.

An important consideration in interpreting the observed variability is that comparative analysis of measured SNVs did not reveal a specific subset that segregates cleanly with high- vs low-infectivity phenotypes; the SNVs identified in the present data set are largely shared between high- and low-infectivity isolates. This observation indicates that the measured SNVs cannot be assigned as causal drivers of infectivity differences among PMSS1-derived isolates, and that some or all may function as passengers whose distribution reflects population-level dynamics—including bottlenecks and selective sweeps during *in vivo* passage—rather than direct causal contributions to phenotype. Furthermore, several classes of genomic variation were not systematically assessed in this study, including recombination and duplication among outer membrane protein genes, phase variation within *hop* genes and lipopolysaccharide biosynthesis loci, and regulatory changes, all of which have been implicated in *H. pylori* colonization capacity and could contribute to the phenotypic differences observed here. Definitive identification of causal genomic determinants would require allelic exchange or complementation in isogenic backgrounds combined with targeted sensitive detection methods (for example, amplicon sequencing) capable of resolving low-frequency variants that may exist below the pooled short-read detection threshold and represents an important direction for future work.

In contrast to PMSS1, we identified PMSS2 as a derivative isolate that is nearly identical to the PMSS1 reference genome and exhibits a high degree of phenotypic stability. Independently isolated PMSS2 single colonies displayed highly consistent colonization levels during both short-term and prolonged mouse infection, providing a genetically homogeneous and phenotypically reproducible experimental system. The availability of PMSS2 thus offers a practical framework for disentangling the effects of pre-existing strain heterogeneity from genomic changes arising during host infection, a distinction that is difficult to achieve when working exclusively with heterogeneous laboratory stocks.

Whole-genome sequencing of single colonies recovered after prolonged infection further revealed contrasting patterns of genomic divergence between PMSS1 and PMSS2. PMSS1-derived colonies recovered from the same mouse remained genetically similar to one another, despite the accumulation of a relatively large number of mutations at the population level. By contrast, PMSS2-derived colonies exhibited greater variability in genetic distances, with individual colonies diverging along distinct genetic trajectories despite a lower overall mutational burden. These observations underscore that the total number of mutations accumulated during infection does not necessarily correlate with the degree of divergence among individual clones and highlight the importance of considering both the quantity and distribution of genomic changes when interpreting infection-associated evolution. This pattern is compatible with the possibility that pre-existing population heterogeneity in PMSS1 may buffer the response to *in vivo* selection, whereas an initially uniform population such as PMSS2 must accumulate new variants during infection to explore comparable phenotypic space.

Re-infection experiments with selected PMSS1-derived isolates further illustrate the complexity of linking genomic variation to infection phenotypes. Low-infectivity isolates were capable of exhibiting increased colonization upon subsequent infection, whereas a high-infectivity isolate maintained robust colonization capacity across infection cycles. These findings suggest that mouse infectivity phenotypes among PMSS1-derived isolates are not uniformly fixed and may be influenced by host-associated pressures during infection. The recoverable colonization capacity observed among initially low-infectivity isolates indicates that reduced infectivity is not necessarily a fixed genetic defect and is compatible with the observation that the bulk PMSS1 stock maintains robust infectivity despite containing individual clones of variable colonization capacity. However, given the limited scope of these experiments, we interpret these observations cautiously, as they may reflect reversible phenotypic responses or differential expression of pre-existing variation rather than stable, genetically fixed adaptation.

Histopathological examination of gastric tissues from mice infected with PMSS1 or PMSS1-derived single-colony isolates revealed comparable degrees of mononuclear cell infiltration at 1 and 4 weeks post-infection, with no significant differences in the severity of gastric lesions between the *H. pylori* PMSS1 strain and PMSS1-derived single-colony isolates (Fig. S5). Within these infection windows, the intrinsic population heterogeneity of PMSS1 thus manifests primarily as variability in colonization efficiency rather than as differences in overtly detectable inflammatory pathology. Whether the population-level heterogeneity described here influences chronic inflammatory or pathological outcomes at extended time points represents an important direction for future investigation.

Together, our findings emphasize the importance of explicitly accounting for strain heterogeneity when using PMSS1 as a model for *H. pylori* infection. While PMSS1 remains a valuable and biologically relevant strain, its intrinsic population diversity introduces variability that can complicate experimental interpretation, particularly in studies aimed at defining host-driven genomic changes. In this context, PMSS2 provides a complementary experimental tool that enables more controlled investigation of infection-associated processes starting from a homogeneous genomic background. More broadly, our results highlight how routine laboratory propagation can shape the genomic landscape of bacterial strains and underscore the need for careful consideration of population structure when drawing conclusions about microbial adaptation *in vivo*.

### Practical recommendations for future PMSS1-based infection studies

Building on the observations described here, we suggest several practical considerations for researchers using PMSS1 or related mouse-colonizing *H. pylori* strains. First, periodic whole-genome sequencing-based characterization of PMSS1 working stocks is valuable to document population composition prior to key infection experiments. Second, the number of laboratory passages between stock revivals should be documented and, where possible, limited to reduce the accumulation of variants that may reduce infectivity. Third, single-colony purification of PMSS1 stocks should be undertaken with caution, as indiscriminate subcloning can inadvertently select derivatives with reduced infectivity; where clonal uniformity is desired, the infectivity phenotype of the derived clone should be verified before use in downstream experiments. Fourth, PMSS2, described in this study, provides a genetically homogeneous companion strain for experiments in which population uniformity of the input inoculum is desirable. Finally, we note the limitation that only male C57BL/6J mice were used in this study; sex-related differences in *H. pylori* colonization and inflammatory responses have been reported, and confirmatory studies including female mice would strengthen the generalizability of our observations.

## MATERIALS AND METHODS

### Bacterial strains and culture conditions

*H. pylori* strain PMSS1, corresponding to the clinical isolate 10700 (30), was used as the parental strain in this study. The parental PMSS1 stock was received in 2017 from the laboratory of Dr. Jay V. Solnick (University of California, Davis) and maintained at −80°C between uses. Prior to the experiments described here, the stock had undergone approximately 10 passages. In this study, working stocks derived from single-colony isolation were expanded through four passages on solid medium before cryopreservation, and pre-infection inocula were prepared by four additional passages (two on solid medium followed by two in Brucella broth), resulting in approximately 18 total passages from original receipt to experimental use. Bacteria were routinely cultured on Columbia agar plates supplemented with 5% defibrinated horse blood and antibiotics (vancomycin, 10 μg/mL; polymyxin B, 0.33 μg/mL; amphotericin B, 5 μg/mL; and trimethoprim, 5 μg/mL) under microaerobic conditions at 37°C. Liquid cultures were grown in Brucella broth (Kisan Bio, Seoul, South Korea) supplemented with 10% fetal bovine serum (Gibco, Grand Island, NY, USA) and 10 μg/mL vancomycin (Duchefa, Haarlem, the Netherlands) for 24 h and were then inoculated into new media to obtain an optical density of 0.05 at 600 nm. These cultures were grown for 18 h with shaking at 120 rpm. All *H. pylori* strains were cultured at 37°C under microaerophilic conditions generated by an Anaeropack-Microaero gas-generating system (Mitsubishi Gas Chemical, Tokyo, Japan). All cultures for a given experiment were prepared in parallel from the same working stock and expanded under identical conditions. For all experiments, bacteria were harvested from plates after 2–3 days of growth unless otherwise specified.

### Single-colony isolation and strain derivation

For single-colony isolation, *H. pylori* PMSS1 cultures were streaked directly from frozen working stocks onto blood agar plates, and well-isolated colonies were selected after 2–3 days of incubation; no liquid-culture revival step was interposed prior to colony picking. A total of 21 PMSS1-derived single colonies (#1–#21) were characterized in this study. Each isolate was independently expanded in liquid culture, and a frozen working stock was prepared at −80°C. For every isolate, the working stock was subsequently re-cultured, and cells from the same culture were split for parallel use: one portion was subjected to genomic DNA extraction for whole-genome sequencing, and a matched portion was used for oral inoculation into mice, so that the sequenced genome and infection phenotype reported for each isolate are directly linked to the same clonal working stock. Whole-genome sequencing was performed on a phenotype-driven subset of 11 isolates representing the two extreme phenotypic classes: four high-infectivity isolates (#2, #5, #18, and #19) and seven low-infectivity isolates (#3, #6, #7, #14, #16, #17, and #20).

During the characterization of PMSS1-derived single-colony isolates, PMSS2 was identified as the high-infectivity isolate #2, a derivative whose genome differed from the PMSS1 reference genome by two SNVs and exhibited high infectivity upon re-infection. To ensure independent strain derivation, PMSS2 was subsequently subjected to the same single-colony isolation procedure as PMSS1, generating a total of 20 independently derived PMSS2 single colonies (#1–#20) for downstream analyses.

### Mouse infection experiments

Male C57BL/6JmsSlc mice (6–8 weeks old, Central Lab. Animal Inc., Seoul, South Korea) were used for infection experiments and were randomly assigned to experimental groups on arrival. Prior to infection, mice were fasted for 18 h and orally inoculated once with approximately 1 × 10^8^ CFU of *H. pylori* suspended in Brucella broth. Two mice were inoculated per bacterial strain or single-colony isolate, and each data point in the figures represents CFU per gram of stomach tissue recovered from an individual mouse. For short-term infection assays, mice were sacrificed 1 week post-infection. For long-term infection experiments, mice were maintained for up to 4 weeks post-infection before analysis.

### Recovery and quantification of *H. pylori* from infected mice

At the indicated time points post-infection, mice were euthanized, and stomachs were aseptically removed. Gastric tissues were opened along the lesser curvature and homogenized in Brucella broth. Serial dilutions of the gastric homogenates were plated onto selective blood agar plates supplemented with antibiotics and incubated under microaerobic conditions at 37°C. CFUs were enumerated after 5–7 days of incubation. For genomic analyses, individual colonies were selected from recovery plates and independently expanded for downstream experiments. Each output single colony was subcultured once on solid medium and then cryopreserved at −80°C to establish an independent frozen working stock. For downstream use, working stocks were revived by patching on solid medium and expanded through one additional passage on solid medium followed by two successive liquid-culture steps in Brucella broth (24 h initial growth, followed by adjustment of the optical density at 600 nm to 0.05 and an additional 18-h incubation) to prepare inocula.

### Re-infection assays

To assess the stability of infection phenotypes across sequential infection cycles, selected output isolates recovered from primary mouse infection experiments were subjected to a secondary *in vivo* passage. Individual colonies from the primary infection recovery plates were picked and expanded to establish independent working stocks, so that each re-infection isolate corresponds to a distinct clonal derivative of the primary output rather than to a pooled culture. These single-colony-derived working stocks were revived and expanded *in vitro* according to the same pre-infection expansion protocol as the primary infection and were re-inoculated into naive mice using the same oral infection protocol described above (approximately 1 × 10^8^ CFU per mouse, matching the primary infection inoculum). At 1 week post-infection, gastric colonization levels were quantified by CFU enumeration. Colonization efficiencies of re-inoculated output isolates were compared with those observed during the primary infection to evaluate the reproducibility and stability of infectivity phenotypes across infection cycles.

### Genomic DNA extraction

Single colonies recovered from mice were cultured for DNA extraction. For pooled samples, all bacteria on the culture plates were collected by swabbing after CFU counting, excluding the colonies selected as individual single colonies. Genomic DNA was extracted using the i-genomic BYF DNA Extraction Mini Kit (iNTRON Biotechnology, Seongnam, South Korea) according to the manufacturer’s instructions, including centrifugation at 16,000 × *g*. DNA purity and concentration were measured using an Epoch Microplate Spectrophotometer (BioTek, Winooski, VT, USA).

### Library preparation and sequencing

Sequencing libraries were prepared using the NEBNext Ultra II FS DNA Library Prep Kit (New England Biolabs, Ipswich, MA, USA). Whole-genome sequencing was performed at Macrogen, Inc. (Seoul, South Korea) using the Illumina NovaSeq 6000 platform. The samples were sequenced with 100 or 150 bp paired-end reads.

### Bioinformatics analysis and variant filtering

Reads were aligned to the PMSS1 reference genome (RefSeq accession: NZ_CP018823.1) using BWA-MEM (v0.7.17), and alignments were further processed with SAMtools (v1.15.1).

The identification of genetic variants for heatmap analysis was performed using two different methodologies depending on the sample type. For the single-colony heatmap analysis, BCFtools (v1.15.1) was utilized to identify single nucleotide polymorphisms (SNPs) between individual isolates. These variants were called without the application of additional allele frequency filters to maintain the raw genetic differences between the single colonies.

In contrast, for the 4-week pooled output population heatmap and evolutionary analysis, variant calling was conducted using FreeBayes (v1.3.10) with specific AF filtering criteria. A threshold of AF ≥10% was applied to the pooled data sets to capture within-host diversification and evolutionary shifts. For direct comparisons between multiple pooled data sets, a more sensitive threshold of AF ≥5% was employed. For identification of variants specific to the mouse-output populations (Fig. 7B), candidate variants were required to have an allele frequency <1% in the corresponding input sample (effectively absent within the detection threshold) and >20% in the pooled output populations of both PMSS1 and PMSS2. The <1% threshold was applied to exclude variants already present in the input population, whereas the >20% threshold was used to minimize background sequencing noise and retain variants that reproducibly expanded during *in vivo* colonization. Additionally, variant loss in the output population was defined strictly as cases of complete extinction (AF = 0).

Functional annotation of all identified SNPs and indels was performed using SnpEff (v5.2f) integrated within the Snippy framework.

### Principal component analysis

Genome-wide SNVs and indels identified across all sequenced samples were converted into PLINK binary format (.bed, .bim, and .fam files), and principal component analysis was performed using PLINK v1.9. The resulting eigenvectors were imported into R (v4.5.1) and visualized using the ggplot2 package. Because the principal component analysis was based on genome-wide variant profiles, the resulting clustering patterns reflect overall genetic similarity among isolates rather than the absolute number of accumulated mutations.

### *cagA* copy number analysis

*cagA* copy number was quantified using two orthogonal approaches. In the sequencing depth-based approach, mean read depth over the *cagA* locus was normalized to the genome-wide mean read depth after mapping short reads to the PMSS1 reference genome (NZ_CP018823.1) using BWA-MEM, and depth values were computed with SAMtools depth. In the quantitative real-time PCR approach, *cagA* copy number was determined by comparative Ct analysis using primers specific to *cagA* and *ureA* (a single-copy reference gene). Concordance between the two methods was evaluated by Pearson correlation. Details of primer sequences and PCR conditions have been described previously (15, 25).

### Histopathological analysis

For histopathological analysis, gastric tissues from infected and uninfected control mice were fixed in 10% neutral buffered formalin, embedded in paraffin, and sectioned. Sections of the gastric corpus and antrum were stained with hematoxylin and eosin and examined by light microscopy. All sections were reviewed and scored in a blinded manner by a co-author with veterinary pathology expertise (DVM).

### Statistical analysis

Statistical analyses were performed using GraphPad Prism v10 (GraphPad Software, La Jolla, CA, USA). For comparisons of CFU/g values among PMSS1-derived single-colony groups (Fig. 2), CFU/g values were log_10_-transformed, and infectivity groups were defined relative to the median CFU/g of the parental PMSS1 stock (1.17 × 10^6^ CFU/g): high infectivity (median ≥1.17 × 10^5^ CFU/g, i.e., within 10-fold of the PMSS1 median), middle infectivity (median between 1.17 × 10^4^ and 1.17 × 10^5^ CFU/g), and low infectivity (median <1.17 × 10^4^ CFU/g). Because CFU/g values were not normally distributed, comparisons among the three infectivity groups were performed using the Kruskal–Wallis test followed by Dunn’s multiple comparisons *post hoc* test. For pairwise comparisons of parental PMSS1 or PMSS2 with derived single-colony groups (Fig. 5), the Mann–Whitney *U* test was used. For comparisons between infection time points (1 vs 4 weeks post-infection), two-way ANOVA on log_10_-transformed CFU/g values was performed followed by Sidak’s multiple comparisons test. Where group sizes were too small to support formal inferential testing (e.g., two mice per single colony in Fig. S3), this limitation is stated in the corresponding figure legend, and results are presented descriptively. Statistical significance was defined as *P* < 0.05.

## Data Availability

The whole-genome sequencing data generated in this study have been deposited in the NCBI Sequence Read Archive (SRA) under BioProject accession number PRJNA1426936, with individual run accession numbers SRX32275308–SRX32275328. These data are publicly available as of the date of publication. Sample-level next-generation sequencing quality metrics for all samples analyzed in this study, including total reads, Q30 rate, GC content, mapping rate, and average sequencing depth, are provided in Table S5.

## SUPPLEMENTAL MATERIAL

Supplemental legends spectrum.01128-26-s0001.docx Legends for Tables S1 to S5.

Supplemental figures spectrum.01128-26-s0002.pptx Fig. S1: Experimental design for single-colony infection assays using PMSS1. Fig. S2: cagA copy number variation among PMSS1 and PMSS1-derived isolates. Fig. S3: Changes in mouse infectivity among PMSS1-derived isolates upon re-infection. Fig. S4: Accumulation of genomic variants in PMSS1 and PMSS2 during mouse infection. Fig. S5: Gastric histopathology at 1 and 4 weeks post-infection with Helicobacter pylori PMSS1 or PMSS1-derived single-colony isolates.

Table S1 spectrum.01128-26-s0003.xlsx SNV and indel variants identified across all sequenced samples, with genomic coordinates, reference/alternate alleles, locus tag, gene name, product, and functional pathway, predicted amino acid change, SnpEff functional impact category, and per-sample allele frequencies. Sheets 1-3: PMSS1-derived single colonies; 4-week output samples; variants plotted in Figure 8.

Table S2 spectrum.01128-26-s0004.xlsx Read depth at each identified variant position for individual samples. Sheets 1-3: PMSS1 laboratory stock; 4-week output samples (individual colonies and pooled output); PMSS1-derived single-colony isolates.

Table S3 spectrum.01128-26-s0005.xlsx Per-sample sequencing depth data used to derive whole-genome sequencing depth-based cagA copy number estimates for the PMSS1 laboratory stock and PMSS1-derived single-colony isolates (Fig. S2).

Table S4 spectrum.01128-26-s0006.xlsx Per-sample sequencing depth data used to derive whole-genome sequencing depth-based cagA copy number estimates for single-colony isolates recovered from mouse stomachs 4 weeks post-infection with PMSS1 or PMSS2 (Figure 6B).

Table S5 spectrum.01128-26-s0007.xlsx Sequencing quality metrics for all samples analyzed in this study—total reads, bases, Q30, GC content, mapping rate, average read length, and average depth.

ASM does not own the copyrights to Supplemental Material that may be linked to, or accessed through, an article. The authors have granted ASM a non-exclusive, world-wide license to publish the Supplemental Material files. Please contact the corresponding author directly for reuse.

## References

[1] Marshall BJ, Warren JR. 1984. Unidentified curved bacilli in the stomach of patients with gastritis and peptic ulceration. Lancet 1:1311–1315. doi:10.1016/s0140-6736(84)91816-6 6145023

[2] Correa P. 1992. Human gastric carcinogenesis: a multistep and multifactorial process--first American cancer society award lecture on cancer epidemiology and prevention. Cancer Res 52:6735–6740. doi:10.1056/NEJMoa001999 1458460

[3] Uemura N, Okamoto S, Yamamoto S, Matsumura N, Yamaguchi S, Yamakido M, Taniyama K, Sasaki N, Schlemper RJ. 2001. *Helicobacter pylori* infection and the development of gastric cancer. N Engl J Med 345:784–789. doi:10.1056/NEJMoa001999 11556297

[4] Peek RM, Blaser MJ. 2002. *Helicobacter pylori* and gastrointestinal tract adenocarcinomas. Nat Rev Cancer 2:28–37. doi:10.1038/nrc703 11902583

[5] Suerbaum S, Achtman M. 2004. *Helicobacter pylori*: recombination, population structure and human migrations. Int J Med Microbiol 294:133–139. doi:10.1016/j.ijmm.2004.06.014 15493823

[6] Morelli G, Didelot X, Kusecek B, Schwarz S, Bahlawane C, Falush D, Suerbaum S, Achtman M. 2010. Microevolution of *Helicobacter pylori* during prolonged infection of single hosts and within families. PLoS Genet 6:e1001036. doi:10.1371/journal.pgen.1001036 PMC2908706 20661309

[7] Kennemann L, Didelot X, Aebischer T, Kuhn S, Drescher B, Droege M, Reinhardt R, Correa P, Meyer TF, Josenhans C, Falush D, Suerbaum S. 2011. *Helicobacter pylori* genome evolution during human infection. Proc Natl Acad Sci USA 108:5033–5038. doi:10.1073/pnas.1018444108 PMC3064335 21383187

[8] Didelot X, Nell S, Yang I, Woltemate S, van der Merwe S, Suerbaum S. 2013. Genomic evolution and transmission of *Helicobacter pylori* in two South African families. Proc Natl Acad Sci USA 110:13880–13885. doi:10.1073/pnas.1304681110 PMC3752273 23898187

[9] Ailloud F, Didelot X, Woltemate S, Pfaffinger G, Overmann J, Bader RC, Schulz C, Malfertheiner P, Suerbaum S. 2019. Within-host evolution of *Helicobacter pylori* shaped by niche-specific adaptation, intragastric migrations and selective sweeps. Nat Commun 10:2273. doi:10.1038/s41467-019-10050-1 PMC6531487 31118420

[10] Watanabe T, Tada M, Nagai H, Sasaki S, Nakao M. 1998. *Helicobacter pylori* infection induces gastric cancer in mongolian gerbils. Gastroenterology 115:642–648. doi:10.1016/s0016-5085(98)70143-x 9721161

[11] Suerbaum S, Michetti P. 2002. *Helicobacter pylori* infection. N Engl J Med 347:1175–1186. doi:10.1056/NEJMra020542 12374879

[12] Salama NR, Hartung ML, Müller A. 2013. Life in the human stomach: persistence strategies of the bacterial pathogen *Helicobacter pylori*. Nat Rev Microbiol 11:385–399. doi:10.1038/nrmicro3016 PMC3733401 23652324

[13] Hatakeyama M. 2004. Oncogenic mechanisms of the *Helicobacter pylori* CagA protein. Nat Rev Cancer 4:688–694. doi:10.1038/nrc1433 15343275

[14] Odenbreit S, Püls J, Sedlmaier B, Gerland E, Fischer W, Haas R. 2000. Translocation of *Helicobacter pylori* CagA into gastric epithelial cells by type IV secretion. Science 287:1497–1500. doi:10.1126/science.287.5457.1497 10688800

[15] Jang S, Su H, Blum FC, Bae S, Choi YH, Kim A, Hong YA, Kim J, Kim JH, Gunawardhana N, Jeon YE, Yoo YJ, Merrell DS, Ge L, Cha JH. 2017. Dynamic expansion and contraction of *cagA* copy number in *Helicobacter pylori* impact development of gastric disease. mBio 8:e01779-16. doi:10.1128/mBio.01779-16 PMC5358911 28223454

[16] Akopyants NS, Clifton SW, Kersulyte D, Crabtree JE, Youree BE, Reece CA, Bukanov NO, Drazek ES, Roe BA, Berg DE. 1998. Analyses of the *cag* pathogenicity island of *Helicobacter pylori*. Mol Microbiol 28:37–53. doi:10.1046/j.1365-2958.1998.00770.x 9593295

[17] Dorer MS, Fero J, Salama NR. 2010. DNA damage triggers genetic exchange in *Helicobacter pylori*. PLoS Pathog 6:e1001026. doi:10.1371/journal.ppat.1001026 PMC2912397 20686662

[18] Draper JL, Hansen LM, Bernick DL, Abedrabbo S, Underwood JG, Kong N, Huang BC, Weis AM, Weimer BC, van Vliet AHM, Pourmand N, Solnick JV, Karplus K, Ottemann KM. 2017. Fallacy of the unique genome: sequence diversity within single *Helicobacter pylori* strains. mBio 8:e02321-16. doi:10.1128/mBio.02321-16 PMC5358919 28223462

[19] Kang J, Blaser MJ. 2006. Bacterial populations as perfect gases: genomic integrity and diversification tensions in *Helicobacter pylori*. Nat Rev Microbiol 4:826–836. doi:10.1038/nrmicro1528 17041630

[20] Andersson DI, Hughes D. 2009. Gene amplification and adaptive evolution in bacteria. Annu Rev Genet 43:167–195. doi:10.1146/annurev-genet-102108-134805 19686082

[21] Elliott KT, Cuff LE, Neidle EL. 2013. Copy number change: evolving views on gene amplification. Future Microbiol 8:887–899. doi:10.2217/fmb.13.53 23841635

[22] Kraft C, Suerbaum S. 2005. Mutation and recombination in *Helicobacter pylori*: mechanisms and role in generating strain diversity. Int J Med Microbiol 295:299–305. doi:10.1016/j.ijmm.2005.06.002 16173496

[23] Cao Q, Didelot X, Wu Z, Li Z, He L, Li Y, Ni M, You Y, Lin X, Li Z, Gong Y, Zheng M, Zhang M, Liu J, Wang W, Bo X, Falush D, Wang S, Zhang J. 2015. Progressive genomic convergence of two *Helicobacter pylori* strains during mixed infection of a patient with chronic gastritis. Gut 64:554–561. doi:10.1136/gutjnl-2014-307345 PMC4392222 25007814

[24] Blaser MJ. 1997. Heterogeneity of *Helicobacter pylori*. Eur J Gastroenterol Hepatol 9 Suppl 1:S3–6; doi:10.1007/978-94-009-1792-7_3 9160209

[25] Jang S, Hansen LM, Su H, Solnick JV, Cha JH. 2022. Host immune response mediates changes in *cagA* copy number and virulence potential of *Helicobacter pylori*. Gut Microbes 14:2044721. doi:10.1080/19490976.2022.2044721 PMC8928821 35289715

[26] Dyer V, Brüggemann H, Sörensen M, Kühl AA, Hoffman K, Brinkmann V, Reines MDM, Zimmerman S, Meyer TF, Koch M. 2018. Genomic features of the Helicobacter pylori strain PMSS1 and its virulence attributes as deduced from its *in vivo* colonisation patterns. Mol Microbiol 110:761–776. doi:10.1111/mmi.14123 30230643

[27] De Maio N, Shaw LP, Hubbard A, George S, Sanderson ND, Swann J, Wick R, AbuOun M, Stubberfield E, Hoosdally SJ, Crook DW, Peto TEA, Sheppard AE, Bailey MJ, Read DS, Anjum MF, Walker AS, Stoesser N. 2019. Comparison of long-read sequencing technologies in the hybrid assembly of complex bacterial genomes. Microb Genom 5:e000294. doi:10.1099/mgen.0.000294 PMC6807382 31483244

[28] Southern EM. 1992. Detection of specific sequences among DNA fragments separated by gel electrophoresis. Biotechnology 24:122–139. 1422006

[29] Cover TL. 2016. *Helicobacter pylori* diversity and gastric cancer risk. mBio 7:e01869–15. doi:10.1128/mBio.01869-15 PMC4742704 26814181

[30] Lee A, O’Rourke J, De Ungria MC, Robertson B, Daskalopoulos G, Dixon MF. 1997. A standardized mouse model of *Helicobacter pylori* infection: introducing the Sydney strain. Gastroenterology 112:1386–1397. doi:10.1016/s0016-5085(97)70155-0 9098027

