# Supplementary material for: Population heterogeneity in Helicobacter pylori PMSS1 shapes variable mouse infectivity: derivation of the homogeneous reference strain PMSS2
Source: Microbiol Spectr. 2026 Sep 17;14(10):e01128-26. doi: 10.1128/spectrum.01128-26 (PMC13689314; doi:10.1128/spectrum.01128-26)
Supplement: Supplementary file 1 [file spectrum.01128-26-s0001.docx]

**Supplementary Table 1. Comprehensive list of SNV and indel variants identified across sequenced samples.**

For each variant, the table reports: (i) genomic coordinate on the PMSS1 reference (NZ_CP018823.1); (ii) reference and alternate alleles; (iii) the nearest coding sequence and its gene name where applicable; (iv) the predicted amino acid change; (v) the SnpEff-annotated functional impact category (High/Moderate/Low/Modifier); and (vi) the sample-by-sample allele frequency across all sequenced isolates and pools. Sheet 1 contains variants identified in PMSS1-derived single colonies; Sheet 2 contains variants identified in 4-week output samples; Sheet 3 contains variants used for the lollipop plot (Figure 8) with annotations of principal virulence factors.

**Supplementary Table 2. Read depth values at variant positions.**

Read depth at each identified variant position is provided for individual samples. Sheet 1 contains depth values for the PMSS1 laboratory stock sample; Sheet 2 contains depth values for 4-week output samples (PMSS1 and PMSS2 individual colonies and pooled output); Sheet 3 contains depth values for PMSS1-derived single-colony isolates. Depth values are provided in addition to the sample-level mean depth summary shown in Supplementary Table 5.

**Supplementary Table 3. Sequencing depth data supporting *cagA* copy number estimation in the PMSS1 laboratory stock and PMSS1-derived single-colony isolates.**

Per-sample sequencing depth values used to derive whole-genome sequencing (WGS) depth-based *cagA* copy number estimates shown in Supplementary Figure 2, for the PMSS1 laboratory stock (PMSS1 LS) and PMSS1-derived single-colony isolates (#2, #3, #5, #6, #7, #14, #16, #17, #18, #19). Columns report: genome depth, the mean per-base sequencing coverage across the PMSS1 reference genome (NZ_CP018823.1); *cagA* depth, the mean per-base sequencing coverage over the *cagA* locus; *cagA* depth/genome depth, the ratio of *cagA* to genome-wide coverage; calibration, the calibrated *cagA* copy number obtained by scaling this ratio to account for the tandem multi-copy architecture of *cagA* in the PMSS1 reference; *vacA*, the mean per-base sequencing coverage over the *vacA* locus (used as a single-copy reference); and *vacA* depth/genome depth, the ratio confirming single-copy occupancy of *vacA*.

**Supplementary Table 4. Sequencing depth data supporting *cagA* copy number estimation in 4-week mouse output single-colony isolates.**

Per-sample sequencing depth values used to derive whole-genome sequencing (WGS) depth-based *cagA* copy number estimates for individual single-colony isolates recovered from mouse stomachs 4 weeks after infection with PMSS1 or PMSS2, as summarized in Figure 6B. Sample identifiers "M1 C1", "M1 C2", "M2 C1", and "M2 C2" denote Colony 1 or Colony 2 isolated from mouse 1 (M1) or mouse 2 (M2) for each of the two parental strains. Columns are defined as in Supplementary Table 3.

**Supplementary Table 5. Quality metrics of next-generation sequencing data used in this study.**

Summary statistics of Illumina NovaSeq 6000 whole-genome sequencing for all bacterial samples analyzed in this study. Sample identifiers follow the convention: "PMSS1 LS" denotes the parental PMSS1 laboratory stock; "PMSS1 single colony #n" denotes independently isolated PMSS1-derived single colonies numbered as in Figure 2; "PMSS1/PMSS2 4w M1/M2 pool" denotes pooled bacterial populations recovered from stomach homogenates of mouse 1 or mouse 2 (M1/M2) at 4 weeks post-infection with PMSS1 or PMSS2; and "M1 C1", "M1 C2", "M2 C1", and "M2 C2" denote individual output single colonies (Colony 1 or Colony 2) isolated from each mouse. Columns report the following metrics: Total Reads, the number of paired-end reads obtained per sample; Bases (Gb), the total number of sequenced bases in gigabases; Q30 (%), the percentage of bases with a quality score of at least Q30; GC Contents (%), the observed GC content of the sequenced reads; Mapping Rate (%), the percentage of reads successfully aligned to the PMSS1 reference genome (NZ_CP018823.1); Average Read Length, the mean sequenced read length in base pairs; and Average Depth (x), the mean per-base sequencing depth across the genome.
